# Impact of internal female migration on unmet need for modern contraception in Zambia

**DOI:** 10.1186/s12978-019-0803-9

**Published:** 2019-11-15

**Authors:** Melanie T. Almonte, Caroline A. Lynch

**Affiliations:** Faculty of Epidemiology and Public Health, London School of Hygiene and Tropical Medicine, London, UK

**Keywords:** Zambia, Migration, Family planning, Modern contraceptive use, Unmet need

## Abstract

**Background:**

Unmet need for contraception, the proportion of women who want to limit or delay childbirth but use no form of contraception, is the core indicator to evaluate the effectiveness of family planning programs. Understanding how migration influences unmet need is important to identify to whom and how to target sexual and reproductive health programs. We assessed how migration status in rural and urban settings is associated with having an unmet need for family planning.

**Methods:**

Data on sexually active, fecund, reproductive-aged (15–49 years) women from the 2013–14 Zambia Demographic and Health Survey were analysed through univariate and multivariate logistic regression models.

**Results:**

Unmet need for modern contraceptive methods was significantly higher among rural to rural migrant women (OR 1.30, 95%CI 1.00–1.70 *p* < 0.05) and rural non-migrant women (OR 1.41, 95%CI 1.06–1.85 *p* < 0.01) compared to urban non-migrant women after controlling for age, marital status, parity, religion, education and wealth.

**Conclusion:**

Women residing in, and migrating between, rural areas were significantly more likely to have an unmet need for contraception. Our findings highlight the importance of understanding migration and migrant streams to strengthen family planning programs. In Zambia, a focus on rural-rural migrants, rural non-migrants and the poorest could improve the health of the entire population.

## Plain English summary

There is limited evidence about how migrating impacts women's family planning. This study aims to address this gap in research by assessing how different migration patterns affect the chances for women to have their reproductive needs (i.e. contraception) unfulfilled (i.e. unmet). An estimated 225 million women in low- and middle-income countries want to avoid pregnancy but use no form of modern contraception (e.g. condoms, the pill). Women at highest risk are typically young, poor, of low education and unmarried, the same population most likely to migrate. Women account for roughly half (48%) of the worlds’ migrants, yet there is a gap in knowledge around how migrating affects women’s ability to plan their families by limiting or delaying future childbirth. In this study, we analysed data from the 2013–2014 Zambia Demographic and Health Survey and compared the chances of having an unmet need for contraception between migrant women and rural non-migrant women to that of urban non-migrant women. We found that rural-to-rural migrants were 30% more likely to have an unmet need and rural non-migrants were 40% more likely compared to urban non-migrants. Regardless of migration status, poor rural women were most likely to have an unmet need compared to rich urban women. In Zambia, a focus on rural-rural migrants, rural non-migrants and the poorest could improve the health of the entire population. More research is needed to improve our understanding of factors that both obstruct and facilitate contraceptive use and how they may be affected by migration over time.

## Background

One billion of the world’s population are migrants of which an estimated 48% are women [1, 2], the majority of whom reside in low- to middle- income countries [3, 4]. Yet, there is limited evidence around how migrating affects women’s ability to plan their families by limiting or delaying future childbirth.

Unmet need for contraception is highest among young, unmarried, and poorly educated nulliparous women, who are also the population most likely to migrate internally [5–9]. The consequences of unsatisfied need for contraceptives include higher incidences of unplanned pregnancies and unsafe abortions which are directly correlated with increased maternal mortality and under-five child mortality rates [10–14]. Therefore, satisfying unmet need is not only vital for the health of women, but also for their children and the wider community.

Yet, limited research is available on the effect of migration on unmet need. Rather, previous studies have focused on its effect on contraceptive use, for which results vary. In Myanmar, Kenya and China, researchers found strong evidence of increased Modern Contraceptive Prevalence (MCP) in rural-to-urban migrants compared to non-migrants, whereas in Cambodia no difference was found [15–18]. In Zambia, access to modern contraception was significantly lower among rural women compared to those living in urban areas [19].

The aim of this study was to understand the effect of internal female migration on unmet need for modern contraception among women in Zambia. Specifically, we examined how different migration pathways may influence women to have an unmet need. Given the evidence that migrant women tend to utilise modern contraceptives and sexual and reproductive health services more than their non-migrant counterparts [15, 16, 19, 20], we attempted to determine whether a change in residence influences the likelihood of unmet need.

## Methods

### Study setting

Internal migration in Zambia is driven by the push and pull of labour market forces rather than insecurity [21]. Urban-urban and rural-rural migration are the dominant migration patterns [22]. In the 1980s, like many other sub-Saharan nations, the government of Zambia implemented land resettlement projects to redistribute the population and wealth by promoting urban-rural migration [23, 24]. As recently as 2017, the Zambian government was renewing efforts to encourage urban-rural migration [25].

In recent years, Zambia made headway towards MDGs 4 & 5, improving maternal and child health respectively. From 2007 to 2013, the under-five mortality rate dropped from 119 per 1000 live births to 87.4 and maternal mortality dropped from 591 per 100,000 live births to 224, however, these results are still unacceptably high when close to 50% of maternal deaths in Zambia could be averted through modern contraceptive use [21, 26–28]. Women in rural areas of Zambia tend to use fewer contraceptives compared to their urban counterparts [29–31]. Access to modern contraceptives in rural areas of Zambia is plagued by a myriad of structural barriers including stock-outs, unavailability of preferred methods and weak provider capacity [29, 32, 33]. The recent push towards ruralisation coupled with poor access to sexual and reproductive health services in rural areas contributes to the importance of better understanding the needs of migrant women [19].

### Data sources and methodology

A secondary analysis of cross-sectional survey data from the 2013–14 Zambia DHS Program was performed for this study. The DHS are openly available nationally representative surveys designed to provide current estimates of demographic and health indicators including fertility, maternal and child health, sexual and reproductive health. Data were collected from eligible women aged 15–49 years by trained interviewers over an 8-month period from August 2013 to April 2014. The sampling frame consisted of a list of provinces broken down into smaller administrative units containing an average of 510 people [29]. This study was limited to married and unmarried women at risk of pregnancy: reproductive age (15–49 years), sexually active and fecund. Sexually active women reported having had sex with at least one person within 3 months prior to the interview [34]. Women who last had sex “before their last birth” were classified as not sexually active since their sexual activity could not be quantified.

### Conceptual framework

A conceptual framework was developed to better identify a priori variables that could influence both unmet need and migration. The framework took into account three key theories that could explain how migration affects fertility, these are: disruption, selection and adaptation theory [35–37]. Disruption theory suggests a migrant’s fertility is temporarily disrupted when spouses are separated, reducing fertility below that of non-migrants. Selection theory proposes that migrants are self-selecting and possess certain characteristics, such as adaptability and risk-taking, and are thus motivated differently compared to non-migrants. Finally, adaptation theory suggests that migrants change their fertility preferences to match that of their host community to meet financial and social constraints [35–37]. These theories work in tandem; therefore it is important to determine the impact of each individually to understand their consequences on contraceptive use [38].

#### Outcome variable

The outcome of interest was unmet need for modern contraception. We applied the DHS Definition for unmet need described by Bradley et al because it is comparable over time and across countries [39]. By this definition, women were considered to have an unmet need if they did not use modern contraceptives, yet were sexually active, fecund and either undecided or did not wish to become pregnant within the next 2 years. Women who have an unmet need for contraception were divided into traditional (e.g. rhythm method, withdrawal) and modern (e.g. the pill, implants, condoms) contraception users. As the latter are more reliable and effective [40, 41], sexually active women using traditional methods were considered to have an unmet need for a more effective modern method [39, 42]. All other women who did not meet these conditions were considered to have a ‘met need’ or ‘no unmet need’. Levels of unmet need were created based on the thresholds defined in several studies [43–48]. We defined Low unmet need as ≤15%, Medium 15.1 to 20%, High 20.1 to 25% and Very High as ≥25.1%.

#### Exposure variables

The primary exposure of interest was migration status: A migrant was defined as one who relocated from one DHS-defined administrative unit to another and has lived in their current residence for at least 3 months at the time of interview; ‘non-migrant’ or ‘internal migrant’. We further divided migration status into four migrant streams based on the direction of movement: non-migrant urban, non-migrant rural, urban-rural migrant, rural-urban migrant. We generated migration variables based on the following questions in the ZDHS regarding the geography and timing of respondents’ moves: type of current residence (rural or urban), years in current residence (range from 0 to 41 years) and type of previous residence (village, town, other city, Lusaka). Non-migrants were defined as having never moved from their current residence. Current residence was delimited according to the cluster in which the interview took place. Years in current residence were the number of years the respondent reported living in the domicile where they were interviewed. Type of previous residence was defined by the respondent’s own classification of their previous residence; *Lusaka*, *other city* and *town* were classified as ‘urban’, and *village* as ‘rural.’ We corroborated this ‘urban’ grouping by combining the proportion of residents in *Lusaka, other city* and *town* with those living in urban areas over the census years 1980, 1990, 2000 and 2010 [29].

Additionally, we sought to determine whether the effect of migration on unmet need is modified by age in that the youngest (15–19 years) and oldest (40–49 years) women would be more likely to experience unmet need compared to non-migrants of the same age. Both age groups have been linked with a low desire to become pregnant, lower modern contraceptive use and underreporting of sexual activity associated with the social stigma of unmarried intercourse, hence would be at higher risk of having unmet need [29, 32, 49].

### Covariates

**Fig. 1:**
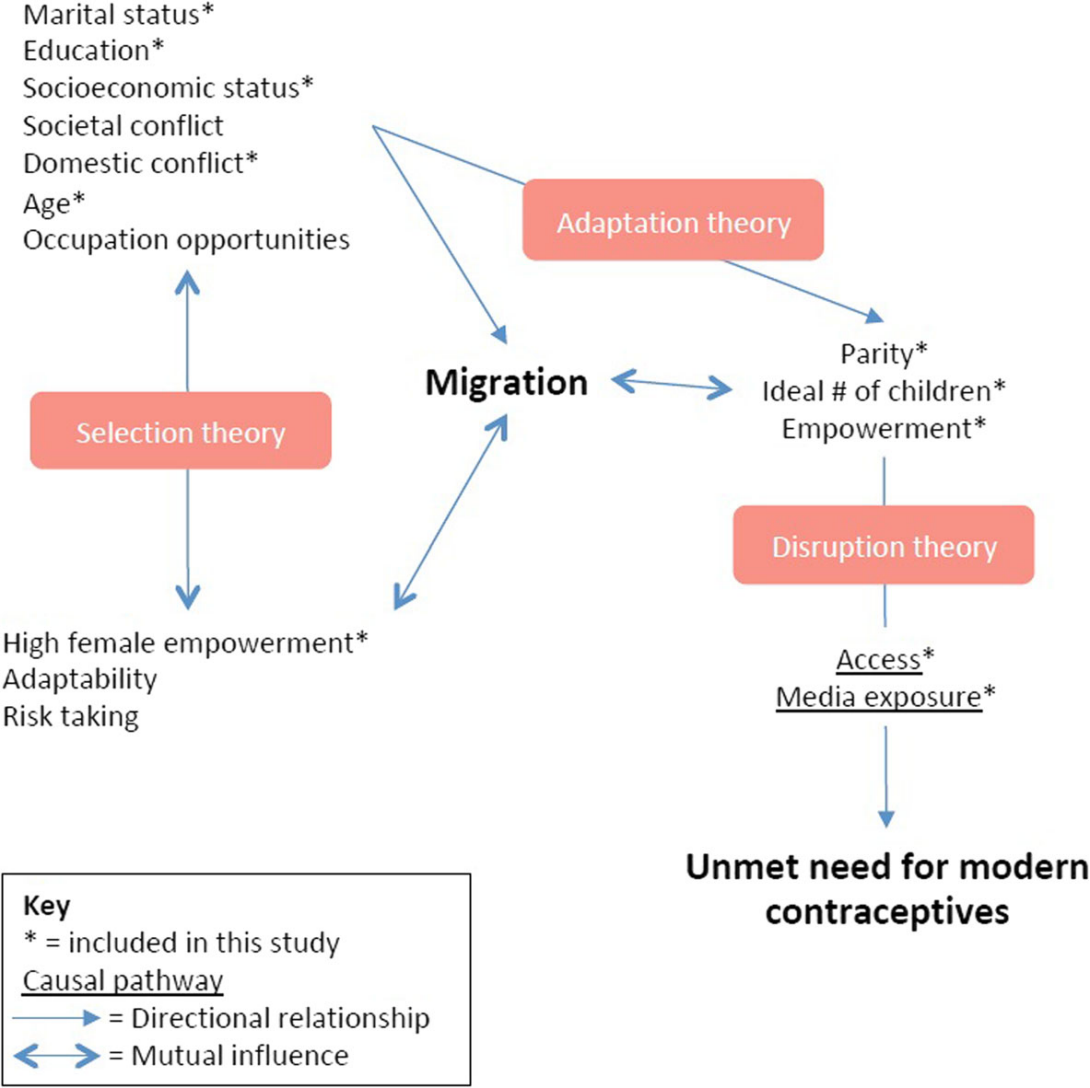
Conceptual framework of the effect of migration on contraception

We included covariates from our conceptual framework that had previously been identified as being strongly associated with migration and/or modern contraceptive use in the literature [15–18] (Fig. 1). Relevant variables available in the ZDHS were adapted and organised into demographic, socio-economic and socio-cultural categories. Demographic variables were: (a) current residence*;* (b) age group; (c) marital status; (d) religion; (e) ethnicity and (f) region. Socioeconomic variables were: (g) wealth index; (h) occupation and (i) education level. Wealth index was created by the DHS and indicates level of wealth in quintiles. It measures a household’s wealth as a composite of household assets, construction material, and water and sanitation variables [29].

Sociocultural variables were measured by: (j) parity; (k) ideal number of children; (l) family planning media exposure and (m) access to modern contraception. A woman with access to modern contraception responded ‘yes’ to either having been visited by a family planning worker or told about contraception at a health facility within the past 12 months.

Empowerment encompasses a wide breath of variation that differs by culture, context and age [50, 51]. Therefore, to adequately control for potentially confounding effects of empowerment, we included two variables. We measured female empowerment via two opinion-based indices included in the DHS: (n) ability to refuse sex, and (o) spousal abuse justified [52, 53]. To measure respondents’ opinion on the ability to refuse sex, respondents answered ‘yes’ or ‘no’ if they agreed that a wife was justified in refusing sex for the following reasons: (i) husband has an STI, (ii) husband has sex with other women or (iii) wife tired or not in the mood. Women who answered ‘yes’ to all scenarios were coded as ‘yes,’ which indicated high empowerment. Women who answered ‘no’ to at least one of these scenarios were classified as ‘no’ indicated low empowerment. To measure respondents’ opinion on whether spousal abuse is justified, respondents answered ‘yes’ or ‘no’ if they believed that a husband was justified in beating his wife for the following reasons (i) refusing sex, (ii) going out without telling husband, (iii) neglecting the children, (iv) arguing with husband, or (v) burning the food. Women who answered ‘no’ to all scenarios were coded as ‘no,’ which indicated high empowerment. Women who answered ‘yes’ to at least one of these scenarios were classified as ‘yes’ indicating low empowerment.

### Analysis

We performed a descriptive analysis with Pearson chi-squared tests comparing unmet need for modern contraception in migrants versus non-migrants, exclusively and within urban and rural settings. Additionally, we compared potential covariates stratified by unmet need. Using logistic regression, we tested each exposure and covariate measure for an association with our outcome. To determine the association between migration status and unmet need, we compared rural non-migrants, rural-urban migrants and urban-rural migrants to urban non-migrants. First, univariate models established crude odds ratios for the effect of migration on unmet need. Covariates that showed strong evidence of an association (*p* < 0.05) with both migration and contraceptive outcomes in the univariate analyses were considered potential confounders. Second, the confounding effects of covariates of interest were examined by creating multivariate models employing a forward stepwise regression. Third, potential confounders were retained in the final multivariate model. Finally, given the association between age and migration explained previously, we fitted an interaction term for these two variables. To measure the potential effect of an unmeasured confounder, such as adaptability of an individual (see Fig. 1), a sensitivity analysis was performed using the E-Value measure based on the potential outcomes framework further described by VanderWeele and Ding (2017) [54, 55]. All analyses were performed using Stata, Release 14 (Stata Corp., College Station, Texas, USA) and were weighted to account for the varying sampling fraction by administrative unit and 3.8% survey non-response rate.

## Results

### Descriptive results

The weighted sample size consisted of 7868 women, 47.9% of the original ZDHS sample (*n* = 16,411). The average age was 30.2 years, 84% were married or had a regular sexual partner, 71% were internal migrants and 57% rural. Just over half of women used modern contraceptives (56%), and almost a quarter (24%) had an unmet need for modern contraception (Table 1). Migrants were more likely to be married (87% vs. 75%) and were exposed to contraception messages in the media (40% vs. 32%).

**Table 1:** Distribution of socio-demographic characteristics in the sample (*n* = 7868, weighted)

*Covariates*	%	n	*Migrant %*	*Non-migrant %*
Current residence				
Rural	56.6	4265	55.4	59.3
Urban	43.4	3603	44.6	40.8
Age, years; mean, (SE)	*30.1 (0.1)*		*30.1 (0.1)*	*29.8 (0.2)*
15–19	10.0	814	8.5	13.4
20–29	40.4	3186	41.6	37.8
30–39	35.4	2743	36.1	33.9
40–49	14.2	1125	13.9	15.0
Marital status				
Unmarried	16.6	1417	12.9	25.2
Married	83.4	6451	87.1	74.8
Religion				
Catholic	17.3	1356	17.1	17.8
Protestant	81.7	6442	81.7	81.6
Musilim/other	1.0	70	1.2	0.6
Ethnicity				
Bemba	22.9	1835	21.9	25.3
Tonga	15.4	964	19.0	6.9
Lozi	5.8	518	5.4	6.7
Other	56.0	4551	53.8	61.1
Region				
Central	9.0	679	8.8	9.6
Copperbelt	15.2	763	13.5	19.0
Eastern	12.8	1023	14.4	9.1
Luapula	6.9	760	5.2	10.8
Lusaka	19.4	898	19.8	18.6
Muchinga	5.2	692	5.2	5.1
Northern	7.4	765	6.9	8.5
North Western	4.1	723	3.5	5.5
Southern	14.3	941	18.1	5.5
Western	5.7	624	4.5	8.4
Parity, mean (SE)	*3.7 (0.4)*		*3.7 (0.6)*	*3.7 (0.7)*
0 to 2	9.0	781	7.7	11.8
1 to 3	44.1	3360	45.6	40.4
4+	47.0	3727	46.6	47.8
Total, % (n)	100.0	7868	71.4% (5616)	28.6 (2252)
Ideal number of children, mean (SE)	*4.9 (0.4)*		*4.9 (0.1)*	*5.0 (0.1)*
0 to 2	7.6	587	5.9	6.7
3 to 4	38.7	3025	40.2	35.3
5+	50.3	3996	49.6	52.0
Non-numeric	3.4	260	2.3	6.1
Wealth				
Poorest	17.6	1368	15.5	22.7
Poorer	18.9	1546	18.2	20.5
Middle	20.5	1806	21.4	18.4
Richer	21.5	1689	23.0	18.0
Richest	21.5	1459	22.0	20.4
Occupation				
Not working	41.3	3027	41.4	41.0
Labourer	30.7	2544	29.5	33.5
Professional	28.0	2297	29.2	25.5
Highest education				
None	8.8	685	8.5	9.6
Primary	51.5	4025	50.7	53.3
Secondary	34.2	2714	34.3	34.0
Higher	5.5	444	6.5	3.1
FP media exposure				
No	62.5	4842	60.0	68.4
Yes	37.5	3026	40.0	31.6
Access to FP				
No	62.5	4864	61.3	65.3
Yes	37.5	3004	38.8	34.7
Empowerment				
Ability to refuse sex				
No	51.9	3949	51.3	53.3
Yes^a^	44.6	3650	45.3	43.2
Don’t know	3.5	269	3.5	3.5
Spousal abuse justified				
No^a^	51.0	3791	52.9	46.3
Yes	45.7	3850	43.7	50.5
Don’t know	3.3	227	3.4	3.2
Total, % (n)	100.0	7868	71.4% (5616)	28.6 (2252)

^a^Denotes high empowerment

Migrants tended to be better off financially than non-migrants; a higher proportion of non-migrants were classified as part of the lowest/poorest (22.7%) quintile than migrants (15.5%). Non-migrants and migrants held comparable views on empowerment; just over half believed that women could not refuse sex under at least one circumstance (53% non-migrant, 51% migrant).

Unmet need was high among all women (23.9%) and was only slightly less prevalent in migrants (23.2%) than non-migrants (25.5%). Unmet need increased with rurality; rural non-migrants bore the highest unmet need (31.3%). More migrants used modern contraceptives (58.4%) than non-migrants (50.5%) with the highest prevalence found in urban-urban migrants (66.2%). Rural non-migrants reported the lowest modern contraceptive prevalence where less than half (44.3%) reported use. Urban non-migrants had a much lower mean parity (mean 2.9) compared to rural non-migrants (mean 4.4) and ideal number of children was high across the board at 5.0 children (Table 2). Finally, unmet need was less common in urban residents (25.4%) than rural residents (29.5%) and less common in the richest women (15.4%) compared to the poorest women (32.5%) regardless of migration or residency (Table 3). Surprisingly, 44.4% (*n* = 8) of non-migrant rural women in the richest wealth quintile reported an unmet need, however we attribute this large proportion to small numbers of women (*n* = 18) who fit this definition.

**Table 2:** Proportion of unmet need for modern family planning within the context of fertility indicators (n = 7868, weighted)

*Migration type*	*Unmet need for modern method*	*Modern contraceptive prevalence*	*Mean parity*	*Mean ideal number of children*	*n (%)*
Migration status	**%**	**%**			
Non-migrant	Very High / 25.5	50.5	3.7	5.0	2252 (28.6)
Migrant	High / 23.2	58.4	3.7	4.9	5616 (71.4)
Migration stream					
Non-migrant urban	Medium / 17.1	59.4	2.9	4.5	848 (10.8)
Urban-urban	Medium / 17.5	66.2	3.0	4.3	2015 (25.6)
Rural-urban	High / 21.2	58.8	3.5	4.8	740 (9.4)
Non-migrant rural	Very High / 31.3	44.3	4.4	5.5	1404 (17.8)
Rural-rural	Very High / 28.1	51.6	4.4	5.4	2206 (28.0)
Urban-rural	High / 23.8	58.5	3.8	4.8	655 (8.4)
Total mean	High / 23.9	56.0	3.7	4.9	7868 (100)

**Table 3:** Proportion of unmet need for modern family planning in migrant and non-migrant women by wealth (n = 7868, weighted)

Wealth quintile						
	*Poorest*	*Poorer*	*Middle*	*Richer*	*Richest*	*Mean row %*
Current Residence						
Rural	32.5	27.0	29.0	20.3	22.9	29.5
Urban	37.7	26.5	16.2	21.1	15.0	25.4
Migration status						
Non-migrant	37.3	27.1	24.3	24.7	12.6	28.3
Migrant	29.6	27.0	25.9	19.6	16.6	25.5
Migration stream						
Non-migrant urban	41.6	28.9	13.9	24.8	11.4	27.3
Urban-urban	18.7	21.1	14.8	19.9	16.3	18.6
Rural-urban	42.8	28.1	21.8	18.9	20.3	27.9
Non-migrant rural	37.2	27.0	28.4	24.3	44.4	29.2
Rural-rural	30.4	27.4	29.1	21.5	21.0	27.1
Urban-rural	23.0	25.6	30.1	14.9	16.1	23.4
All women in sample	32.5 (450/1368)	27.0 (431/1546)	25.5 (437/1806)	20.9 (340/1689)	15.4 (248/1459)	24.2 (1906/7868)

### Analysis results

Univariate analysis showed that unmet need for contraception was significantly higher among both non-migrant rural women (OR 2.21, 95%CI 1.70–2.86) and urban-rural migrants (OR 1.51, 95%CI 1.10–2.09) compared to urban non-migrant women (Table 4). Unmet need was highest among women who resided in rural areas (OR 1.84, 95%CI 1.60–2.12), were aged 40–49 years (OR 1.66, 95%CI 1.33–2.07), were unable to numerically verbalise their ideal number of children (OR 2.37, 95%CI 1.59–3.54), believed in a religion other than Protestant or Catholic (OR 1.86, 95%CI 1.08–3.21) and believed that spousal abuse was justified (OR 1.29, 95%CI 1.13–1.48). When the confounding effects of age, marital status, parity, wealth and education were controlled for in our multivariate model, the odds of having an unmet need for contraception was reduced in rural non-migrant women, yet they still experienced 40% more unmet need (OR 1.40, 95%CI 1.06–1.86) compared to urban non-migrants (Table 4). We also found evidence that rural-rural migrant women were 30% more likely to experience unmet need (OR 1.30, 95%CI 0.99–1.70) compared to urban non-migrants. Yet, urban-rural (OR 1.16, 95%CI 0.83–1.60) and rural-urban (OR 1.11, 95%CI 0.80–1.54) migrants experienced an almost identical likelihood of unmet need compared to urban non-migrants. Regarding potential effect modification, evidence of an interaction between age and migration and unmet need was not detected (not shown). Results of the sensitivity analysis indicate that an unmeasured confounder would have to be associated with both migration and unmet need by an odds ratio of 1.19-fold each to explain the lower confidence limit, meaning little additional unmeasured confounding is necessary to explain the effect observed.

**Table 4:** Association between unmet need for modern family planning and migration stream and covariates in women aged 15–49 years in Zambia

*Independent variable*	Univariate model		Multivariate model	
	*Odds ratio*	*95% CI*	*Odds ratio*	*95% CI*
Migration stream [Urban non-migrant]				
Rural non-migrant	2.21***	1.70–2.86	1.41**	1.06–1.85
Rural-rural migrant	1.90***	1.47–2.45	1.30*	1.00–1.70
Urban-rural migrant	1.51**	1.10–2.09	1.16	0.83–1.60
Urban-urban migrant	1.03	0.78–1.35	1.08	0.82–1.42
Rural-urban migrant	1.31	0.95–1.80	1.11	0.80–1.54
Current residence [Urban]				
Rural	1.84***	1.60–2.12		
Age [15–19]				
20–29	0.60***	0.49–0.73	0.62***	0.50–0.78
30–39	0.72***	0.59–0.88	0.61***	0.47–0.78
40–49	1.66***	1.33–2.07	1.29	0.97–1.70
Marital status [Unmarried]				
Married	1.11	0.92–1.33	0.97	0.78–1.21
Religion [Catholic]				
Protestant	1.15	0.96–1.37		
Other	1.86*	1.08–3.21		
Ethnicity [Bemba]				
Tonga	0.97	0.77–1.22		
Lozi	0.99	0.76–1.30		
Other	0.96	0.82–1.12		
Region [Central]				
Copperbelt	0.71**	0.54–0.92		
Eastern	0.80	0.62–1.04		
Luapula	1.18	0.89–1.56		
Lusaka	0.60***	0.45–0.79		
Muchinga	1.40*	1.02–1.93		
Northern	2.01***	1.49–2.72		
North Western	1.14	0.87–1.49		
Southern	0.88	0.67–1.16		
Western	0.83	0.60–1.14		
Parity [0]				
1 to 3	0.69**	0.55–0.87	0.85	0.65–1.11
4+	1.30*	1.03–1.65	1.26	0.92–1.71
Ideal number of children [0 to 2]				
3 to 4	0.91	0.69–1.19		
5+	1.42*	1.07–1.89		
Non-numeric	2.37***	1.59–3.54		
Wealth index [Poorest]				
Poorer	0.77**	0.64–0.92	0.78**	0.65–0.94
Middle	0.71***	0.59–0.85	0.74**	0.61–0.90
Richer	0.55***	0.44–0.68	0.71**	0.56–0.90
Richest	0.38***	0.30–0.47	0.57***	0.43–0.76
Occupation [Not working]				
Labourer	1.61***	1.39–1.87		
Professional	0.86	0.73–1.02		
Highest education [None]				
Primary	0.77*	0.63–0.95	0.89	0.72–1.09
Secondary or higher	0.47***	0.37–0.59	0.80	0.63–1.02
FP media exposure [No]				
Yes	0.70***	0.61–0.79		
Access to FP [No]				
Yes	0.75***	0.65–0.87		
Empowerment				
*Ability to refuse sex [No]*				
Yes	0.83**	0.73–0.95		
Don’t know	1.10	0.79–1.52		
*Spousal abuse justified [No]*				
Yes	1.29***	1.13–1.48		
Don’t know	1.18	0.84–1.65		

* < 0.05, ** < 0.01, *** < 0.001

## Discussion

This study examined the impact of internal migration on unmet need for modern contraception among Zambian women of reproductive age (15–49 years). Our findings provide evidence that rural-rural migrant and rural non-migrant women were more likely to experience unmet need compared to urban non-migrant women. Migrants moving from urban-to-rural areas or in the opposite direction did not have increased odds of unmet need, nor did migrants moving between urban areas.

Findings among women who reside in, and migrate between, rural areas initially suggest that either there is a lack of access to, or availability of, contraceptive services, or low intention to use even in the face of high unmet need. Several studies have drawn attention to the disparity in access between rural and urban Zambia [19, 56–58]. Commonly cited barriers to contraceptive use there include high cost, limited contraceptive choice, distance from services and poor transportation links [16, 59–62]. Yet, women migrating from urban-to-rural areas did not have significantly different unmet need from their non-migrant urban counterparts. This may be a reflection of a variety of factors including wider social acceptability of contraception and greater availability of modern contraceptive methods in urban areas [19, 32], but it does beg the question as to whether women migrating from urban to rural areas are able to prioritise their needs over potential social stigma. Similarly, there was no significant difference in unmet need between women migrating from rural to urban areas and urban non-migrants. Again, this suggests that women moving into urban areas were either more easily able to physically or financially access contraceptives, or that their intention to use changed once they arrived in their new destinations. Overall findings indicate that women adapt to their local circumstances in one direction (rural to urban) but not in the other suggesting that there are factors beyond migration that drive whether women’s unmet need is disrupted or whether she can adapt to new circumstances. Results of the sensitivity analysis indicate that even a weak association between both migration and unmet need with an unmeasured factor could negate the significance of our main outcome. It is possible that unmeasured characteristics of migrant women may include those related to migration theories presented (Fig. 1) e.g. whether a woman adapts new behaviours relative to another or is prepared to take more risks.

It is possible that urban to rural migrants may be wealthier than their rural resident counterparts, meaning they can more easily get to health facilities. Greater wealth and urban residence are often highly correlated [19]. Our results showed a clear pattern between wealth and unmet need, with the likelihood of unmet need increasing with every one-unit decrease in wealth quintile. The poorer a woman is, the more likely she is to have unmet need: the least poor women are 43% less likely to have an unmet need compared to the poorest women in our study. Wealth strongly confounded our results. Previous studies also noted an association between wealth and residence [18, 19]. In this study, the majority (84%) of urban women were classified as being part of the two highest (richest, richer) wealth quintiles whereas only 12% of rural women reached those quintiles.

Irrespective, understanding the potential role that migrants from urban areas could play in influencing women in rural areas may allow programmes to target messages to women with unmet need. One potential role of urban to rural migrants may be to act as ‘champions’ of contraceptive use who actively engage with young, poorer women with highest unmet need [63–65]. This is an especially interesting concept given the Zambian government’s promotion of ruralisation.

Several limitations of this study are attributable to inherent issues using DHS surveys, which aggregate a lifetime of migration history, and are limited in the detail on what they can capture. While we could examine womens long- and short- term migration status, we are unable to draw a causal relationship between migration and unmet need due to the cross-sectional nature of DHS data. In addition, we are unable to examine the heterogeneity of migration streams in detail as the DHS migration module does not include details such as distances travelled, whether they women migrated internally or externally.

Nevertheless, where most previous studies focus on unmet need in married women, we included unmarried women in our study and limited our sample only to married women who reported being sexually active. While social stigma is associated with condom use and promiscuity in Zambia leading to possible underreporting of unmarried women’s sexual behaviour our estimates may better reflect the true odds and prevalence of unmet need in this population and the low investment in contraceptive care [33, 66–68].

A better understanding of the impact of migration on women’s unmet need would be possible through research that can segment migrant streams of women and better understand characteristics associated with those streams. One potential avenue for further research could be to collect data longitudinally on one individual over time through a cohort that captures more detail on women’s migration within Zambia. This would enable researchers to determine whether unmet need preceded migration or is a consequence of it. Our results raise questions about the motivations and behaviour of migrant women in addressing their unmet needs that would require more qualitative investigation and research into how to determine an individuals’ ‘adaptability’ and risk-taking capacity that may lead them to migrate in the first instance.

## Conclusion

Our findings demonstrate that migration impacts women’s unmet need, and access to contraceptives. However, a better understanding of the motivation and behaviours of women migrating from urban to rural areas and in the opposite direction could allow national programmes to better understand why women residing in, and migrating between, rural areas continue to have unmet needs for contraception.

## Data Availability

The dataset supporting the conclusions of this article are publicly available through the Demographic and Health Services Program repository, https://dhsprogram.com/what-we-do/survey/survey-display-406.cfm.
